# Dynamic System Stability Modeling Approach with Sparrow-Inspired Meta-Heuristic Optimization Algorithm

**DOI:** 10.3390/biomimetics8050424

**Published:** 2023-09-13

**Authors:** Tianqi Xia, Mingming Zhang, Shaohong Wang

**Affiliations:** 1 Fan Gongxiu Honors College, Faculty of Science, Beijing University of Technology, Beijing 100124, China; xiatianqi@emails.bjut.edu.cn; 2 Key Laboratory of Modern Measurement and Control Technology, Beijing Information Science and Technology University, Beijing 100192, China; wangsh78@126.com; 3 Zhengzhou Aerotropolis Institute of Artificial Intelligence, Zhengzhou 451162, China

**Keywords:** dynamic system stability, spatial mode, feature extraction, fuzzy entropy, neural network modeling, sparrow-inspired optimization algorithm

## Abstract

Aiming at the accurate prediction of the inception of instability in a compressor, a dynamic system stability model is proposed based on a sparrow-inspired meta-heuristic optimization algorithm in this article. To achieve this goal, a spatial mode is employed for flow field feature extraction and modeling object acquisition. The nonlinear characteristic presented in the system is addressed using fuzzy entropy as the identification strategy to provide a basis for instability determination. Using Sparrow Search Algorithm (SSA) optimization, a Radial Basis Function Neural Network (RBFNN) is achieved for the performance prediction of system status. A Logistic SSA solution is first established to seek the optimal parameters of the RBFNN to enhance prediction accuracy and stability. On the basis of the RBFNN-LSSA hybrid model, the stall inception is detected about 35.8 revolutions in advance using fuzzy entropy identification. To further improve the multi-step network model, a Tent SSA is introduced to promote the accuracy and robustness of the model. A wider range of potential solutions within the TSSA are explored by incorporating the Tent mapping function. The TSSA-based optimization method proves a suitable adaptation for complex nonlinear dynamic modeling. And this method demonstrates superior performance, achieving 42 revolutions of advance warning with multi-step prediction. This RBFNN-TSSA model represents a novel and promising approach to the application of system modeling. These findings contribute to enhancing the abnormal warning capability of dynamic systems in compressors.

## 1. Introduction

Encountering instability in a compressor may lead to a serious threat to flight safety. It is essential to effectively monitor status data during compressor operation and take appropriate control measures based on the data feedback. By intelligently identifying the preconditions for the compressor state, it is possible to proactively adjust and avoid the occurrence of instability. Researchers have made significant progress in understanding the unstable mechanisms of compressor systems. For instance, the flow instability characteristic was investigated in an experimental and numerical study by introducing a method based on Fourier transform and variation mode decomposition [1]. Due to the complexity of fluid dynamics, there is still a lot of room for improvement.

Entropy, as a nonlinear dynamic measure, is considered to help with capturing the complexity and uncertainty in a system. As a metric quantifying chaos and uncertainty [2], entropy has broad applications, aiding in understanding system properties and behaviors. Various types of entropy have been proposed in time series analysis. With a discussion on the pros and cons of sample entropy and permutation entropy, Rostaghi [3] explored the application of discrete entropy in a time series. Safari [4] introduced different entropy indicators in association with emergence and self-organization for time series classification. Based on these associations, the authors proposed an alternative joint entropy in which each feature was represented using a specific entropy indicator. An improved multi-scale fuzzy entropy method [5] was developed to obtain information about the operation of a centrifugal compressor using empirical mode decomposition. A finite sequence distance was defined based on inversion, and a new entropy method was derived called fuzzy permutation entropy (FPE) [6]. The results indicated that FPE could effectively distinguish deterministic signals from random signals. Subsequently, an improved sample entropy algorithm [7] was proposed based on nonlinear dynamics for identifying precursors of stall instability in axial flow compressors. In the prediction of compressor instability, the concept of entropy can assist in making sense of the dynamic characteristics and stability of the system comprehensively.

However, traditional methods driven by physical models require significant prior knowledge and complex mathematical models, limiting their flexibility and adaptability in practical applications. As a powerful machine learning technique, deep-learning approaches are adopted to learn complex feature representations from large amounts of data and exhibit strong generalization abilities. An adaptive feature fusion learning method [8] was proposed for stall precursor detection and diagnosis based on vibration signals. The model combined multiple-scale features to reduce the complexity of the ensemble model and improve its generalization performance. Quan [9] employed deep neural networks for the accurate detection of stall precursors and achieved fast anomaly detection in a time series. A hybrid neural network model was proposed specifically via the data fusion of spatial mode amplitudes [10]. The established fusion model effectively represented the nonlinear characteristics of a rotating stall.

While a deep-learning network possesses a strong capability for automatically learning complex features and patterns, entropy is adopted to assess the complexity and information of a system in time series data. Chen [11] modeled a post-time series via a deep neural network (DNN) using sample entropy, referred to as SampEn-DNN. An aerodynamic system model with Support Vector Regression was introduced to predict the precursor of a stall optimized using the Gray Wolf algorithm [12]. When utilizing wavelet reconstruction coefficients as input, the proposed hybrid model exhibited better predictive accuracy and reliability. The integration of entropy and deep-learning networks offers a promising improvement in understanding complex systems and carrying out accurate modeling in applications.

The Instability of a system is observed to exhibit remarkable nonlinearity for stall inception. Picking the modeling object is the critical first step to successfully identifying flow characteristics. Because of the complicated mechanism of inception, the nonlinear behavior of the flow is required for quantitative characterization. The logic of entropy analysis is believed to provide a perspective for the identification strategy on stall inception. So, it is shown that researchers must seek a reliable entropy algorithm to quantify the expression status of systems.

Additionally, model selection and parameter optimization are the other issues that require an appropriate network architecture and optimization algorithm. When the model is overly complex or trained on limited data, it may lead to the risk of overfitting. The interpretability of complex deep-learning networks may also be challenging, and they have limited reliability in practical applications [13]. Meta-heuristic algorithms are a class of optimization techniques based on heuristic search, aiming to solve complex parameter optimization. These algorithms imitate heuristic search strategies found in nature to seek global optimal solutions or high-quality solutions for parameter configurations for neural networks [14]. This integration is particularly promising in deep neural network training, as it can avoid suboptimization and expedite the training process. A vision-based crack diagnosis method [15] was developed using a deep convolutional neural network (DCNN) and the enhanced chicken swarm algorithm (ECSA) to detect damaged concrete. Based on stacked autoencoders, an enhanced whale optimization algorithm [16] was applied for the diagnosis of concrete arch beams, providing a satisfying result with limited sensors. A hybrid metaheuristic algorithm named chaotic sand cat swarm optimization [17] was proposed for complex optimization problems to improve global search performance and convergence behavior. These research studies prove that the meta heuristic-optimized neural networks have good potential in engineering applications.

To address these challenges, the fuzzy entropy and the sparrow-inspired optimization algorithm are introduced into the RBF network to model the system stability in this paper. As a measure of disorder, fuzzy entropy can help to identify the complexity and uncertainty of the data. In the recognition of stall precursor, the fuzziness of the state is quantified to determine the instability of the system. The sparrow algorithm, as a heuristic optimization algorithm, is designed to carry out the optimal solutions mimicking the foraging behavior of sparrows. During the optimization process, it can help find the appropriate model parameter configurations, thereby improving the performance and the generalization ability of the stability model. Moreover, when dealing with missing values due to sampling issues, the RBF networks prove to have an advantage capability in estimating the missing values, producing lower mean squared errors compared to other methods [18]. The combined applications of these methods provide reliable and efficient solutions in the modeling of compressor systems.

The main work of this article can be organized in the following aspects. Firstly, the spatial mode analysis is applied for the data processing. The spatial pattern information is extracted to provide a foundation for the subsequent state recognition. For the system parameter characterization, the application of fuzzy entropy is adopted for the detection of the instability precursor with a recognition threshold. Next, the RBFNN is employed for the dynamic system stability prediction model. Using the Logistic SSA optimization, the accurate model can achieve the performance prediction of system status. The approach of the RBFNN-LSSA model significantly improves the early warning capability of instability detection compared to the direct fuzzy entropy analysis. By introducing the Tent mapping into SSA optimization, the prediction ability is further enhanced. The proposed RBFNN-TSSA method shows superior performance, especially in multi-step prediction scenarios. The aim of this article is to enhance the prediction ability of compressor systems with neural network modeling inspired by sparrow optimization, providing an effective tool for relevant applications.

## 2. Modeling Methodology

### 2.1. Fuzzy Entropy Algorithm

Fuzzy entropy [19] is an algorithm used to measure the degree of uncertainty or randomness in a time series. The procedure of fuzzy entropy algorithm is as follows. For a time series X of length N, the phase space reconstruction is performed to obtain a new time series *Y*. Specifically, for each position i, a continuous set of *m* values is taken as the vector Y(i) shown in Equation (1),

(1) $$Y\left(i\right)=\left[x\left(i\right),x\left(i+1\right),\ldots ,x\left(i+m-1\right)\right]-x0\left(i\right),i=1,2,\ldots ,N-m+1.$$

where *m* is the embedding dimension, and $x_{0}\left(i\right)$ is the mean of that vector.

The distance between time series $Y\left(i\right)$ and $Y\left(j\right)$ is defined as $d_{i,j}^{m}$ in the following formula:

(2) $$d_{i,j}^{m}={max}_{k\in \left(0,m-1\right)}\left|\left[x_{i}+k-x0\left(i\right)\right]-\left[x_{j}+k-x0\left(j\right)\right]\right|$$

With introducing the fuzzy membership function, the similarity $D_{i,j}^{m}$ between time series $Y\left(i\right)$ and $Y\left(j\right)$ is calculated as

(3) $$D_{i,j}^{m}=\exp\left(-\frac{(d_{i,j}^{m}{)}^{n}}{r}\right)$$

where r is the similarity tolerance, 1 ≤ j ≤ N=m+1, and i ≠ j.

The function $\psi^{m}\left(r\right)$ is defined as follows:

(4) $$\psi^{m}\left(r\right)=\frac{1}{N-m+1}\sum_{i=1}^{N-m+1}\left(\frac{1}{N-m}\sum_{j=1,j\neq i}^{N-m+1}D_{i,j}^{m}\right)$$

The fuzzy entropy FuzzyEn(m, r) of the original time series is indicated as

(5) $$\begin{array}{ccc} & FuzzyEn(m,r) & =\underset{N\to \infty }{\lim}[\ln(\psi^{m}(r))-\ln(\psi^{m+1}(r))] \end{array}$$

where N is tended to infinity. For the finite-length time series, the estimate of fuzzy entropy is given via

(6) $$FuzzyEn(m,r,N)=\ln(\psi^{m}(r))-\ln(\psi^{m+1}(r))$$

The fuzzy entropy algorithm is an improved sample entropy by introducing a fuzzy membership function. The possibility is measured for the new patterns generated. The complexity of a time series is evaluated by determining the similarity between vectors. A higher value of fuzzy entropy indicates a greater uncertainty of the time series. This algorithm extensively applies in fields such as time series analysis and pattern recognition to understand and quantify the ambiguity of compressor states.

### 2.2. Radial Basis Function Neural Network

The radial basis function neural network (RBFNN) [20,21] is an artificial neural network model consisting of input, hidden, and output layers. The core features of the RBF networks lie in the use of radial basis functions as activation functions in the hidden layers. Firstly, the input layers receive the input vectors and pass them to the hidden layers. Each neuron in the hidden layers is associated with a radial basis function. The activation value of each neuron is computed based on the distance between the input and its corresponding weight vector. Typically, a Gaussian function is used as the following radial basis function:

(7) $$f\left(x\right)=e^{-\frac{(x-\mu {)}^{2}}{2\sigma^{2}}}$$

where x is the input vector, μ represents the center point of the neuron (considered as the weight vector associated with the neuron), and σ is the width parameter of the radial basis function. The activation values in the hidden layers are obtained and passed to the output layers. The output layers are usually linear layers combined with the activation values from the hidden layers to produce the final outputs.

The training process of the neural network involves two key steps: the center point selection and the weight adjustment. For the former one, algorithms such as k-means clustering are used to choose the center points of the hidden layer neurons. In weight adjustment, methods like least squares or gradient descents are employed to optimize the weights between the hidden and output layers.

The RBF neural networks possess strong approximation capabilities for nonlinear problems of high dimensionality and missing data. It is available for applications of pattern recognition, function approximation, and time series analysis.

### 2.3. Sparrow Search Algorithm

The sparrow search algorithm (SSA) [22] is a heuristic optimization algorithm based on the foraging behavior and group cooperation of sparrows. The search behaviors of sparrows during foraging are imitated to solve optimization problems. The principle of the SSA algorithm is firstly to initialize a group of sparrow individuals, with each individual representing a potential solution to the problem. Then, iterative optimizations are performed by imitating the search behavior of sparrows.

In every iteration, each sparrow individual adjusts its position based on the distance between its current position and the best position (global optimal solution), as well as the distance from other sparrow individuals. This position adjustment is performed with the following formula:

(8) $$x_{i}(t+1)=x_{i}(t)+\lambda (t)\cdot \Delta x_{i}(t)$$

where $x_{i}(t)$ is the current position of individual $x_{i}$, $x_{i}(t+1)$ is the position of individual $x_{i}$ in the next generation, λ(t) is the learning rate, and $\Delta x_{i}(t)$ is a randomly generated search step size.

Logistic mapping is a nonlinear mapping function commonly used in chaos theory and dynamic systems. It maps the real number of the domain into a range between 0 and 1 with special dynamic properties. The expression for the logistic mapping is given as

(9) $$x_{n+1}=r\cdot x_{n}\cdot \left(1-x_{n}\right)$$

where $x_{n}$ represents the value at the n iteration, and $x_{n+1}$ represents the value at the next iteration. The control parameter r determines the properties of the mapping. In the sparrow search algorithm, logistic mapping is used to control the randomness and diversity of sparrow individuals during the search process. Typically, the randomness in the SSA is controlled via factors such as the learning rate and search step size. And logistic mapping provides a convenient method for generating random factors. 

Specifically, in the position updated formula of the SSA, the logistic mapping can generate random numbers as a part of the search step size $\Delta x_{i}(t)$. This step increases the randomness of the search, allowing sparrow individuals to explore more extensively in the solution space and avoid getting trapped in local optima. By adjusting the control parameter *r* in the mapping function, the distribution range of the generated random number can be regulated to influence the search performance of the SSA algorithm. The application of logistic mapping in the SSA aims to increase the randomness and diversity of the search. It provides a controllable random generation mechanism to enhance the global search capability and convergence performance.

### 2.4. Error Evaluation Metrics

The mean absolute error (MAE) [23,24] as the performance evaluation metric indicates the quality of solution and the effectiveness of the SSA algorithm in optimization problems. The index of MAE assesses the average of the absolute differences between the predicted and true values. A smaller MAE expresses a higher accuracy of the model, as it implies the predicted value is closer to the true value. The computation for MAE is defined as

(10) $$MAE=\frac{1}{n}\sum_{i=1}^{n}|y_{i}-{\hat{y}}_{i}|$$

where n represents the number of samples, $y_{i}$ represents the true value of the i sample, and ${\hat{y}}_{i}$ represents the predicted value for the i sample.

As the evaluation metric for the objective function, the optimization objective is to minimize the errors between predicted and true values. By assessing the quality of the current solution, appropriate adjustments can be made based on the objective function. Specifically, the fitness of each individual is evaluated in the SSA with MAE. By comparing the fitness values, individuals with better fitness can be selected as the next generation of the population. Through iterative optimization, it gradually approaches the optimal solution. And the variation in MAE is assessed for the quality of solution process to achieve more accurate and robust optimization results.

## 3. Feature Extraction of Research Object

The object of this study is a certain type of low-speed axial flow compressor. The compressor has adopted C4 airfoil blade arrangements, with a designed rotational speed of 3000 rpm and a total pressure rise of 1500 Pa. The detailed introduction to the research object is depicted in the literature [12]. The relevant geometric parameters of the compressor are indicated in Table 1 for a brief vision.

In the experimental study on the instability of the axial flow compressor, five Kulite dynamic pressure sensors are installed on the casing wall of the inlet. These sensors are evenly distributed around the circumference to acquire the static pressure. The static pressure is recorded with a sampling frequency of 5000 Hz via the sensor during the development of stall revolution at the working state. In this study, the instability process of the axial flow compressor is investigated at a speed of 2500 rpm. For the feature extraction involved in the nonlinear data, a data fusion procedure is processed to achieve the spatial mode by a discrete Fourier transform (DFT). The static pressure signals are transformed into the pattern of traveling waves. And the first-order spatial mode is considered to be well enough to reflect the basic characteristics of the nonlinear system [7].

**Figure 1:**
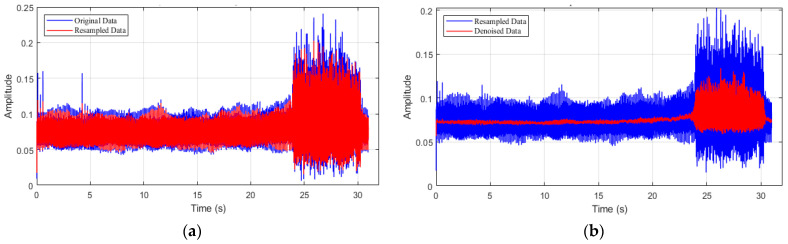
First-order spatial mode of system. (**a**) Resampled data; (**b**) Denoised data.

In the data processing, the sample data of the first-order spatial mode are resampled as 500 Hz. However, the resampling process may introduce additional noise signals, so it is necessary to denoise the resampled data. A local weighted regression technique is employed for this data processing. The resampled spatial mode is revealed in Figure 1, as well as the denoised process.

The evolution process of the compression system behaves with the variation in modal wave energy for entering the unstable state. The fundamental nature of the system can be implied accurately from the sharp increase in the amplitude of modal energy as the state transitions. Then, the boundary of the rotating stall is determined to be the borderline of the stability in the system. From the view of system identification, the energy of spatial mode can be picked as the quantization parameter for instability recognition. Therefore, the modeling of the system stability in the following sections proceeds based on the spatial modal directly for the identification of the stall inception.

## 4. Stability Identification Strategy with Fuzzy Entropy Algorithm

To achieve the identification of the inception of instability, the fuzzy entropy is performed to reflect the difference in the evolution of the compressor state. In the entropy computation in spatial mode, a sliding window with step size is set based on the algorithm definition. The selections of the window parameters may affect the accuracy of fuzzy entropy on identification. So, the width of the window should be discussed first. A large window size would result in containing a long time span of information for each window and lead to small changes in the fuzzy entropy between the windows. The fluctuation sensitivity is reduced, which is not conducive to the recognition. On the other hand, a significant impact is shown on the overall fuzzy entropy fluctuation with the small window size. This could weaken the recognition accuracy on the instability boundary by noise error.

**Figure 2:**
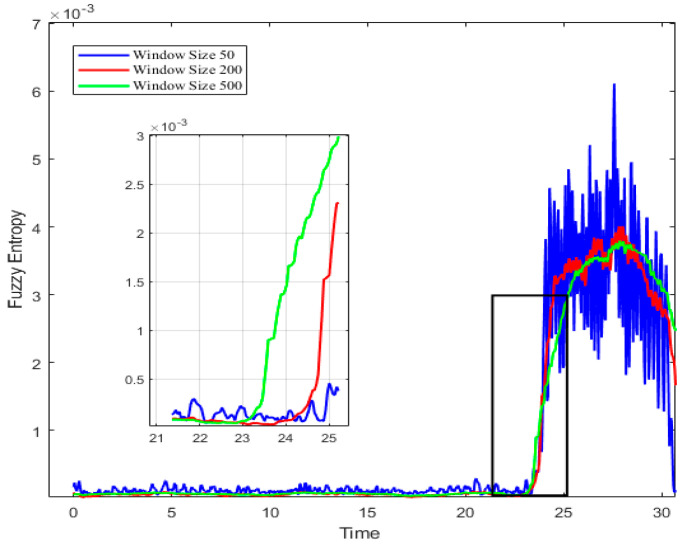
Fuzzy entropy with sliding window size.

In the fuzzy entropy algorithm, the embedding dimension *m* is set to be 2, and the similarity tolerance *r* is equal to $0.2\;Std(W_{i})$, where $Std(W_{i})$ represents the standard deviation of the window data $W_{i}$. The variations of fuzzy entropy are indicated in Figure 2, with different window widths at 2500 rpm. According to the suggestion on the parameter selection [25], the sliding step size of 20 and the sliding window size of 200 are chosen here to minimize noise interference and enhance the significance detection. Appropriate parameters can contribute to analyzing the spatial pattern for the inception recognition. And it can quantitatively distinguish the property at the instability boundary of the system, as the square marked in the chart.

**Figure 3:**
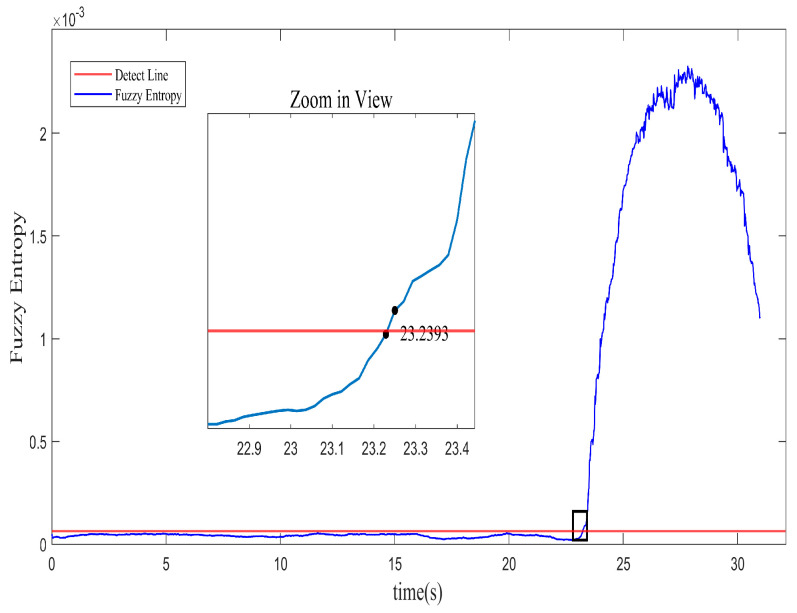
Recognition of location with identification strategy.

The level of entropy reflects the uncertainty and chaotic nature of the system. A higher entropy value indicates a complex and chaotic system, while a lower entropy value indicates an orderly and stable system. In order to accurately identify the system instability location, the identification strategy is designed with the 3σ principle [25]. The recognition threshold ε is placed to be the mean value plus three times the standard deviation. The average entropy under stable conditions is considered the benchmark. The threshold is expressed as

(11) $$\varepsilon =\bar{\omega }+3\sigma ,$$

where the standard deviation is represented as σ, and the mean entropy value in stable conditions is represented as $\bar{\omega }$. If the fuzzy entropy value deviates from the threshold set for recognition, this location is marked as a detection point for system instability. In the normal operating condition, the first-order spatial mode remains relatively stable with small fluctuations. And the changes in fuzzy entropy are minimal, indicating a simple and regular system with low uncertainty. As pointed at the intersection of the detection line and the fuzzy entropy in Figure 3, the inception of system instability labels at approximately 23.2393 s, correspond to 968.3 revolutions. It means that the instability inception of the system can be recognized about 30.35 revolutions in advance using the identification strategy with the fuzzy entropy algorithm.

Approaching the stall boundary, the spatial mode undergoes an extreme wave motion. In the graph, the sudden increase in fuzzy entropy at the unstable region indicates a rise in instability and complexity in the system. The variation in fuzzy entropy for the spatial mode is consistent with the experimental phenomena. It again confirms the feasibility of fuzzy entropy for the prediction of system instability. The detection of instability based on the abrupt change in fuzzy entropy further facilitates the identification of stall phenomena in the compressor.

## 5. System Modeling with Sparrow-Inspired Optimization Algorithm

### 5.1. Modeling of Single-Step Prediction with Logistic Sparrow Algorithm

From the above analysis, the spatial mode reflects the essential flow characteristic of the rotating stall in the system. In this section, the spatial mode is modeled for the dynamic system via the RBF neural networks with the sparrow-inspired optimization algorithm. Firstly, the establishment of the RBFNN model is underway with a single-step solution optimized via the logistic sparrow search algorithm (LSSA). The LSSA is utilized to seek the optimal parameters of the RBFNN for enhancing the prediction accuracy and stability.

There are three parameters to be optimized in the networks the centers of the basis functions, the variances, and the weights of layers. The centers of the basis functions refer to the points in the input space at which the radial basis functions are centered. These centers determine how the network responds to different input patterns. The optimization of the centers involves finding the most suitable locations to accurately represent the data distribution. The variances determine the spread or width of the radial basis functions. It involves finding the appropriate spread of the basis functions to capture the variations presented in the data. The weights connecting the hidden layers to the output layers play a crucial role in mapping the hidden layer’s representations to the desired outputs. It involves adjusting the values to minimize the difference between the network predictions and the actual target values.

**Figure 4:**
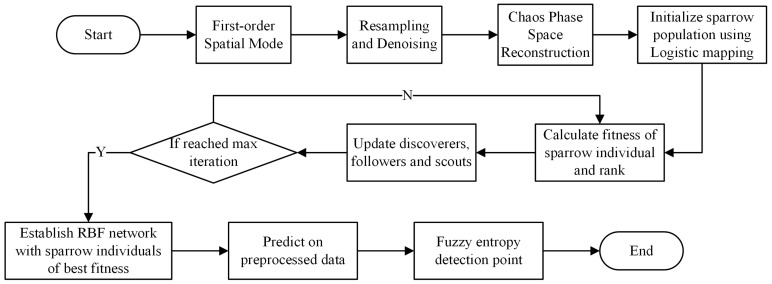
Flowchart of RBFNN model optimized by LSSA.

The optimized RBFNN-LSSA model is employed for the system status prediction subsequently. And the generalization ability is estimated with the test sample data. The predicted result served as a precursor for the instability of the compressor. Furthermore, the stall inception of the compressor is monitored with the fuzzy entropy as the detection strategy. Fuzzy entropy is capable of assessing the complexity and uncertainty of data sequences. The significant variation in fuzzy entropy indicates the index as the occurrence of compressor instability. The modeling process of the RBFNN-LSSA hybrid model is illustrated in Figure 4.

**Figure 5:**
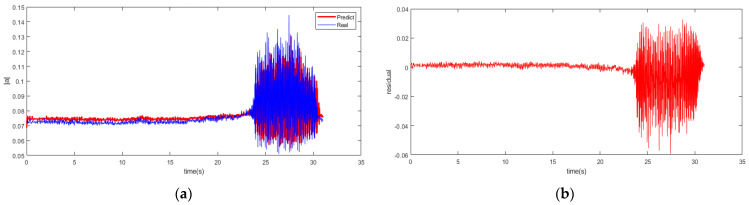
Single-step prediction of RBFNN-LSSA model. (**a**) Prediction profile; (**b**) Residual profile.

The SSA is set up to a population of 20 with a max iteration of 200. The results of the single-step prediction for the spatial mode of the system are indicated in Figure 5 with the RBFNN-LSSA model at 2500 rpm. Although the prediction profile is in existing errors compared to the actual value, the overall reliability and accuracy are still responded with the hybrid modeling for operation state in the system. This indicates that the early precursor of instability of the compressor can be well reflected and captured. The advancement in prediction can enable the detection of subtle changes in the system, thus avoiding potential instability issues.

**Figure 6:**
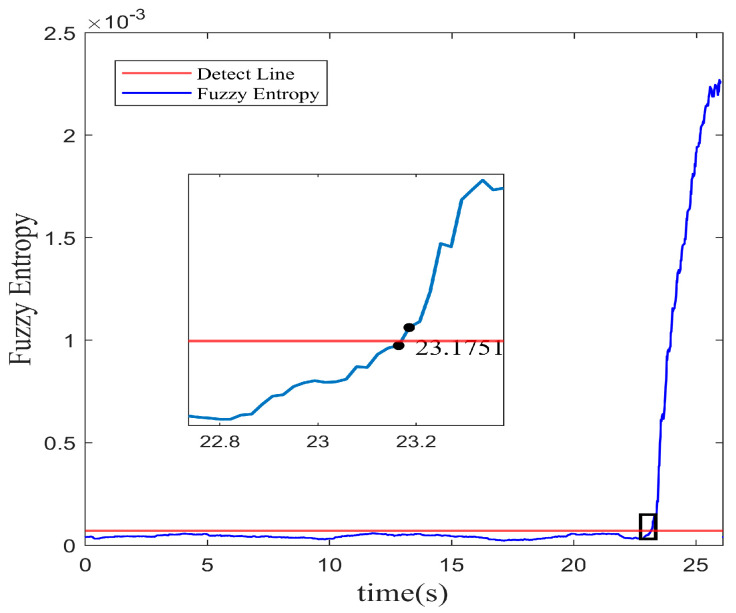
Fuzzy entropy of spatial mode with single-step prediction.

According to the results of the single-step prediction model established by the RBFNN-LSSA method, the instability of the system is identified using the fuzzy entropy algorithm. As represented in Figure 6, the stall inception is marked at approximately 23.175 s with the 3σ principle. Therefore, based on the RBFNN-LSSA hybrid model with single step prediction, the instability of the system can be detected about 33.12 revolutions in advance with the fuzzy entropy analysis.

It indicates that the prediction result from the RBFNN-LSSA model effectively reflects the essential dynamic feature in the system. This similarity provides an accurate and reliable foundation for the fuzzy entropy solution. And it is certificated with the advancement in the detection location of instability. This integration of the intelligence approach presents an effective route for early warning of compressor instability, offering a promising solution for efficient and accurate monitoring in practical applications.

### 5.2. Modeling of Multi-Step Prediction with Logistic Sparrow Algorithm

An early identification of the unstable boundary is of great significance for the safe operation of the compressor. For the purpose of obtaining a larger margin for the inception recognition, the multi-step solution is explored in this section for the system modeling optimized with the logistic sparrow algorithm. The multi-step prediction provides a forecast of the system state over a longer time horizon, which is crucial for the evaluation of the operating state. By continuously predicting multiple time steps ahead, it can gain a comprehensive understanding of the evolution trend in the system, which contributes to identifying potential instability issues. 

**Figure 7:**
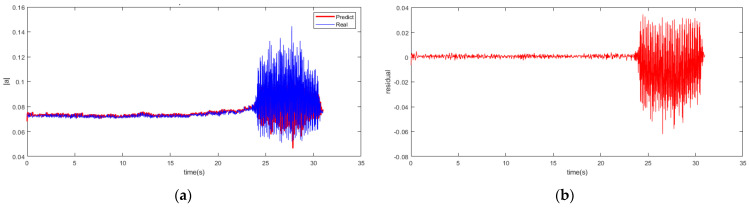
Multi-step prediction of RBFNN-LSSA model. (**a**) Prediction profile; (**b**) Residual profile.

It is worth noting that there would be a certain degree of performance degradation with an increase in prediction step length. So, it is meaningful to select an appropriate prediction step length for the balance between the model accuracy and instability recognition margin. With the examinations of 5/10 prediction multi-steps carried out using the RBFNN-LSSA model, the prediction of the spatial modal amplitude is pictured with the 10 multi-step model in Figure 7, as well as the residual in the process.

**Figure 8:**
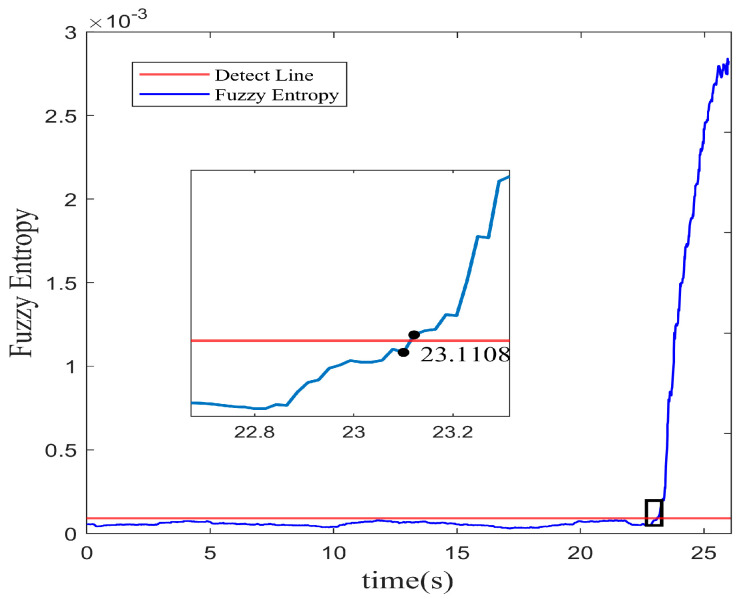
Fuzzy entropy of spatial mode with multi-step RBFNN-LSSA prediction.

It shows that the amplitude predicted is basically consistent with the data of spatial mode in the experiment. The residual error of the multi-step prediction still proves an acceptable precision accuracy within a low range. The profile of fuzzy entropy with 10 multi-step prediction models reveals in Figure 8. The region with a rapid increase in amplitude is regarded as the stall inception of instability in the system. In the same way, the instability recognition threshold is set with the 3σ principle. On the basis of the RBFNN-LSSA hybrid multi-step model, the stall inception is detected at 23.11 s, which corresponds to 35.8 revolutions in advance.

To evaluate the performance of the proposed hybrid model, a comparison is listed in Table 2 with the MAE indicator of the related works. The RBFNN-LSSA model proposed in this exploration reflects an accurate ability for system modeling. The established hybrid network model proves an excellent effectiveness for nonlinear dynamic system modeling. With the LSSA optimization, the accuracy of the dynamic system model can be effectively improved.

### 5.3. Modeling Improvement with the Tent Mapping Sparrow Algorithm

In an effort to further improve the performance of the network model, the sparrow search algorithm attempts to optimize with the tent mapping instead of the logistic mapping. The tent mapping typically has a broader search space, which facilitates a more comprehensive exploration of potential solutions. In contrast, logistic mapping may be limited by its specific mapping function, leading to a relatively smaller search space. Replacing the mapping function potentially enhances the accuracy of system modeling as well as the iteration speed of the neural network.

The tent map is referred to as a real function with a parameter *u*, represented as

(12) $$f_{u}(x)=\min(x,1-x).$$

The parameter *u* in the tent mapping values from 0 to 2. This mapping transforms the interval [0, 1] onto itself, thereby defining a discrete-time dynamic system that can be interpreted as a recursion relationship. The tent mapping function exhibits a topological conjugacy with the logistic mapping. 

**Figure 9:**
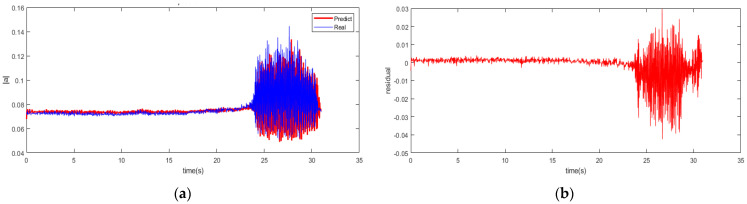
Multi-step prediction of RBFNN-TSSA model. (**a**) Prediction profile; (**b**) Residual profile.

By incorporating the tent mapping function, a wider range of potential solutions within the TSSA is explored, providing flexibility in identifying optimal parameters. In the optimization with a broader space, the multi-step prediction performance is improved for the system modeling. With the same input as Section 5.2, the multi-step predictions are carried out using the RBFNN-TSSA model for the spatial mode of the system. And the results are displayed in Figure 9 with 10 multi-step predictions.

**Figure 10:**
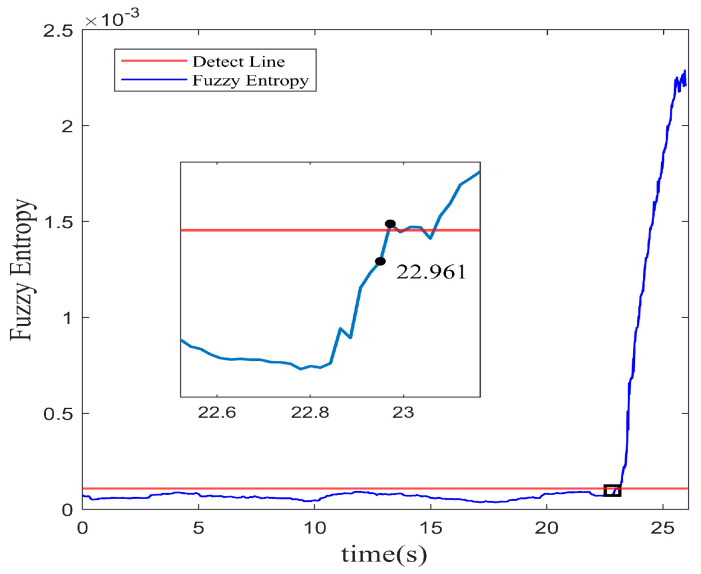
Fuzzy entropy of spatial mode with multi-step RBFNN-TSSA prediction.

From Figure 10, it observes that by using the TSSA, the inception of system instability is recognized at 22.96 s. The early warning signal can be lighted about 42.06 revolutions in advance. It outperforms the LSSA method by shifting an earlier detection of 6.2 revolutions. In multi-step prediction, a wider range of information is involved, so the adaptability of the tent mapping is advantageous in handling the uncertainty and complexity of data. The results indicate that the TSSA-based optimization method demonstrates a suitable adaptability for complex nonlinear dynamic modeling.

Moreover, the TSSA maintains a diverse population and facilitates the global optimal solution during the search process. Due to its better global search ability and genetic property, the tent mapping function shows a faster convergence. Within the given number of iterations, the TSSA has a higher probability of searching the global optimum.

## 6. Discussion

In application, the multi-step predictions acquire a wider range of warning time, which meets the demand of instability identification. In the investigation of this paper, it shows a better modeling accuracy in the single-step solution. But, an earlier warning signal is achieved with the system modeling of a multi-step solution. From this point, the balance between the model accuracy and warning margin is worthy of consideration in the identification of nonlinear system instability.

To measure the effectiveness and applicability of the neural network model, the performances are summarized in Table 3 as comparisons with the related models. The RBFNN fuzzy entropy model is also experimented with chaotic sand cat swarm optimization [17]. Overall, both the logistic and the tent RBFNN-SSA models can accurately provide early warnings on the occurrence of instability in the system. It can be concluded that the proposed RBFNN-SSA models show certain advantages in prediction accuracy and instability inception recognition. With an increase in prediction steps, the forecasting time range is also correspondingly improved. The solutions are computed using an Intel i7-6500U processor, with running times of 11 ms and 13 ms for the single step and 84 ms and 89 ms for 10 multi-step with LSSA and TSSA, respectively. Due to the slight differences in running time, the RBFNN-TSSA model achieves the best behavior of 42 revolutions advance warning with 10 multi-step predictions in the present explorations.

The tent SSA may have several advantages over the logistic SSA. Firstly, in terms of the convergence speed, the tent SSA typically exhibits a faster convergence. Utilizing the characteristic of tent mapping, it can adjust network parameters rapidly to achieve faster optimal solutions. Secondly, by employing tent mapping, the TSSA algorithm can better adapt to the distributions and characteristics of the input data, thereby improving the accuracy and stability of the prediction model. Additionally, the TSSA demonstrates a robustness in handling noise and outliers in the data. And it possesses strong adaptability and generalization capability and has a good predictive performance in potential applications.

Meanwhile, upon the aforementioned discussions, the proposed method possesses several limitations. In-depth Feature Fusion: While the spatial mode is adopted to fuse features of the flow field, a comprehensive exploration of various feature fusions is still insufficient. The effective methods of integrating multiple features could be enhanced for further research.

Single Optimization Algorithm: This study primarily employs the single SSA algorithm for the optimization of the RBF network. A comparative comparison with various optimization methods would provide insights into their strengths and weaknesses in system modeling for further study.

Diversity analysis: The diversity metric is one of the techniques used for balance analysis of the convergence rate and global optimum. It focuses on the population diversity level to reduce the probability of falling into the local optima trap during the search [17]. This analysis is addressed as future research content for the diversity of agents across iterations.

## 7. Conclusions

A dynamic system modeling with a sparrow-inspired optimization algorithm is proposed in this article for the inception prediction of instability in compressors. The application of fuzzy entropy is adopted for the detection of instability with the spatial pattern information. Using the SSA optimization, the RBF neural network achieves the performance prediction of system status. By introducing logistic and tent mapping into SSA, the prediction ability is further enhanced. Using the identification strategy, the instability inception can be detected in advance. The main conclusions can be summarized as follows.

(1) From the view of system identification, the energy of spatial mode can be picked as the quantization parameter for instability recognition. By observing the variation in modal wave energy, the instability can be implied accurately by the sharp increase as the state transitions. The nonlinear characteristic presented in the system is addressed by the fuzzy entropy to reflect the differences in the evolution of the compressor state. Approaching the stall boundary, the sudden increase in fuzzy entropy indicates a rise in complexity in the system. The instability inception can be recognized about 30.35 revolutions in advance using the identification strategy with the fuzzy entropy algorithm.
(2) The establishment of the RBFNN model is carried out with the solutions of the single-step and multi-step predictions. The LSSA is utilized to seek the optimal parameters of the RBFNN to enhance the prediction accuracy and stability. With the optimization of LSSA, the accuracy of the dynamic system model can be effectively improved. On the basis of the RBFNN-LSSA hybrid multi-step model, the stall inception is detected about 35.8 revolutions in advance. In comparison with the related works, the RBFNN-LSSA model proposed in this exploration proves excellent effectiveness for nonlinear dynamic system modeling.
(3) To further improve the network model, the logistic mapping is replaced with the tent mapping to generate the sparrow population. By incorporating the tent mapping function, a wider range of potential solutions within the TSSA is explored to provide flexibility in identifying optimal parameters. The TSSA-based optimization method demonstrates a suitable adaptability for complex nonlinear dynamic modeling. According to the results, the RBFNN-TSSA model achieves the best behavior of 42 revolutions advance warning with 10 multi-step predictions in the present explorations.

It is noticed that biological heuristic optimization algorithms play an increasingly important role in optimal solutions of neural network parameters. There is still room for improvement in neural network modeling, especially for the prediction with multi-steps. The metaheuristic algorithms are worthy of further exploration for upgrading the accuracy of modeling, such as the chicken swarm algorithm, sand cat swarm algorithm, whale optimization algorithm, etc.

## Figures and Tables

**Table 1:** Geometric parameters of the experiment subject [12].

Parameter	Value
Design speed n (rpm)	3000
Outer diameter D (mm)	450
Blade height h (mm)	56
Tip speed (m/s)	70.7
Hub-tip ratio	0.75
Rotor blade number	19
Stator blade number	13

**Table 2:** MAE comparison of the proposed model with related works at 2500 rpm.

Method		MAE
Logistic mapping RBFNN-SSA model	single-step	$8.4\times 10^{-4}$
	5 multi-step	$1.0\times 10^{-3}$
	10 multi-step	$1.1\times 10^{-3}$
Chaos-K-means-GD-RBF model [14]		$1.2\times 10^{-3}$
Chaos–K-means–RBF model [14]		$1.8\times 10^{-3}$

**Table 3:** Performance comparison of the related models at 2500 rpm.

Method		MAE	Early Warning
RBFNN-LSSA fuzzy entropy model	Single step	$8.4\times 10^{-4}$	33.12 revs
	5 multi-step	$1.0\times 10^{-3}$	34.01 revs
	10 multi-step	$1.1\times 10^{-3}$	35.80 revs
RBFNN-TSSA fuzzy entropy model	single-step	$8.6\times 10^{-4}$	32.90 revs
	5 multi-step	$9.7\times 10^{-4}$	34.90 revs
	10 multi-step	$1.1\times 10^{-3}$	42.04 revs
Chaos-K-means-GD-RBF model [14]		$1.2\times 10^{-3}$	12.30 revs
Chaos–K-means–RBF model [14]		$1.8\times 10^{-3}$	-
RBFNN fuzzy entropy model with CSCSO [17]		$1.6\times 10^{-3}$	25.86 revs
Wavelet singular entropy model [25]		-	23.75 revs

## Data Availability

Not applicable.
